# Chinese Pre-service Music Teachers’ Perceptions of Augmented Reality-Assisted Musical Instrument Learning

**DOI:** 10.3389/fpsyg.2021.609028

**Published:** 2021-02-04

**Authors:** Bing Mei, Shuxia Yang

**Affiliations:** ^1^School of Foreign Languages, Henan University, Kaifeng, China; ^2^Institute of Foreign Linguistics and Applied Linguistics, Henan University, Kaifeng, China

**Keywords:** teacher education, augmented reality, musical instrument, perception, China

## Abstract

Given the rapid growth of music technology, this study reports Chinese pre-service music teachers’ perceptions of musical instrument learning assisted by augmented reality (AR). In this study, we conducted a small-scale case study with six pre-service teachers enrolled in a music teacher training programme at a comprehensive university in China. Participants engaged in semi-structured, face-to-face interviews after hands-on experiences with an AR-based piano learning app. Thematic analysis revealed that the participants were generally aware of the potential of this instructional approach but doubted its efficacy and exhibited weak intention to adopt it in their future classrooms. Implications of the findings for music teacher training are discussed.

## Introduction

The educational field has long recognised the importance of music to human development, especially the direct positive influence of music education on the development of children’s cognitive and social abilities (Goble(ed.), 2010; Elliott and Silverman, 2015; Barrett et al., 2019). Therefore, music education is commonly incorporated into educational curricula worldwide (Hallam et al., 2009; McPherson and Welch, 2012; Khalil et al., 2019). Meanwhile, prior studies (McCarthy and Goble, 2002; Bowman, 2003; Regelski and Gates(eds), 2009) also reveal that music education is under the joint influence of shifting economic, social, and cultural realities. With the exponential growth of information and communication technology (ICT) in recent years, the ways to experience music have undergone a noticeable change towards growing reliance on digital methods (King and Himonides(eds), 2016; Tuuri and Koskela, 2020). This is especially the case in an increasingly digitised China (China Internet Network Information Center, 2020). In view of this trend, technology-assisted music education has been embraced by educational policy makers in China, who hope that this initiative will help to reform and reshape music education in the country (Ministry of Education, 2012, 2018). Recently, with the rapid development of augmented reality (AR) technologies, interest has emerged in linking AR to musical instrument learning (Johnson et al., 2020; Martin-Gutierrez et al., 2020). Considering the key role of pre-service music teachers in supporting the initiative of technology-assisted music education (Conway et al., 2019), this study aimed to capture the perceptions of such teachers on AR-assisted musical instrument learning. We hope that our findings will offer new insights for the ongoing exploration of technology-assisted music learning and elucidate the future development of music teacher training programmes in China.

### Music Education in Chinese Schools

As of 2019, China had approximately 245,000 primary and secondary schools and about 45,000,000 students (Ministry of Education, 2020). Thanks to the rapid social, economic, and cultural development over the past four decades, music education in China has progressed significantly (Ho, 2014). It is currently mandated that students at primary and secondary levels should have 1–2 music classes per week. These classes should include music activities such as singing, instrumental learning, music appreciation, music reading, and sight singing (Yu and Leung, 2019). However, in practice, most classes are singing-centred. Further, because music is not a compulsory component of *gaokao* (OECD, 2016), music education is mostly marginalised compared with core subjects such as English, mathematics, and science (Yu and Leung, 2019). Moreover, the content and form of music education in China’s public schools largely depends on the competence of music teachers, parental support, and the economic development of local areas (Xie and Leung, 2011; Yu, 2016). For example, in affluent urban areas, many schools have their own choirs, bands, or dance groups, because these schools can hire well-trained music teachers and parents can afford to send their children for after-school private lessons to learn how to play musical instruments. In rural areas, by contrast, the stress from high-stakes examinations, the cost associated with music education, and the lack of qualified music teachers jointly makes it difficult to guarantee weekly music lessons, let alone regular lessons for musical instruments (Sun and Leung, 2013). In summary, there is a prevailing aspiration for music education but contextual constraints cause considerable variation across schools and between urban and rural areas, which makes meeting the curricular goals of music education a challenging task in practice (Ministry of Education, 2019).

### Music Teacher Education in China

In China, pre-service music teachers usually receive training in the four types of educational institutions: (1) conservatory of music, (2) music schools at normal universities and comprehensive universities, (3) teacher training colleges, and (4) specialised teacher training schools. In general, primary or secondary school music teachers are mostly trained in either Type 2 or Type 3 institutions, with Type 1 institution cultivating music professionals and Type 4 institution serving the pre-school level (Yu, 2016). As for the content of the training programme, there is no official guidance and it tends to vary across institutions. But, in general, to be in line with guidelines from the Ministry of Education (2006, 2011), the curriculum should include the following three core modules: (1) music education ideology (e.g., being familiar with traditional Chinese and classical Western music); (2) musical abilities and practices (e.g., singing, dancing, and playing the piano and other musical instruments); (3) professional capacities (e.g., use of information and communication technology, instructional design, and organising extra-curricular activities).

### AR in Education

AR is a medium that “combines real and virtual objects in a real environment, runs interactively in three dimensions [i.e., physical space] and in real time, and registers (aligns) real and virtual objects with each other” (Azuma et al., 2001, p. 34). With these properties, AR products provide new and enriched ways to see, hear, and feel the world (van Krevelen and Poelman, 2010). Extant research (Zhou et al., 2008; Montero et al., 2019) suggests that based on the technical feature of AR applications, AR technologies roughly fall into three categories: (1) marker-based AR (e.g., QR codes); (2) sensor-based AR (e.g., geolocation sensors); and (3) model-based AR (e.g., optical character recognition models). Currently, existing research findings suggest that AR-based education is still in its infancy and some prevalent challenges such as dependence on hardware and software (Wu et al., 2013), lack of AR-based educational resources (Parmaxi and Demetriou, 2020), and presence of technical problems (Sirakaya and Alsancak Sirakaya, 2018) have been identified. However, the rapid advancement in mobile technologies has enabled AR to step out of research labs and become accessible on users’ mobile devices. With a growing list of AR applications, users can enjoy the enriched experiences afforded by AR without being constrained to a specially equipped area (Cipresso et al., 2018). Thus, the potential of AR has drawn the attention of educational researchers and practitioners. Empirical studies have examined the pedagogical potential and effectiveness of AR in teaching a variety of subjects, such as science, mathematics, environment, and language (Radu, 2014; Jain et al., 2017; Lee and Park, 2019). Recent findings indicate that AR-assisted teaching and learning activities provide a face-to-face collaborative learning environment that effectively enhances learners’ social interactivity and fosters a strong sense of team spirit (Lin et al., 2015). It has also been noted that the learning activities designed with AR could effectively increase learners’ interest in the topic, improve their learning outcomes, and promote their thinking and investigation skills (Wu et al., 2013; Akçaylr et al., 2016).

Thanks to rising research focus on using AR for educational purposes, studies on AR-assisted music education have also started to gain momentum. For example, Lemos et al. (2017) evaluated the effectiveness of teaching musical notes to children with an Android app called *AR Musical*. Their findings reveal that this app can keep learners highly engaged in the learning activity and provoke their motivation and interest, which in turn help participants gain accurate knowledge of the learning content. Further, the audio and visual feedback provided by the app through AR technology can stimulate multiple learning channels simultaneously and help to develop young learners’ visual (Di Serio et al., 2013), auditory (Bauer et al., 2019), and motor skills (Chang et al., 2020).

In sum, our literature review suggests that linking ICT to music education is a promising research topic. With the popularisation of smartphones, ubiquitous access to the Internet, and AR’s capacity to provide spontaneous feedback, AR-assisted music education seems to be a viable approach to alleviate the shortage of qualified music teachers and enhance music education in China. However, it has long been noted by educational researchers (e.g., Kagan, 1992) that teachers’ classroom practices are highly influenced by their beliefs. In the context of China, music teachers’ views of technology-assisted music education remain little understood. To address this gap, the current study focused on Chinese pre-service music teachers. It aimed to answer the following research question:

How do Chinese pre-service music teachers perceive AR-assisted musical instrument learning?

## Methods

### Design

Considering that the AR-assisted education is at its liminal stage (e.g., Kang, 2018; Cook, 2019; Johnson et al., 2020) and there were practical constraints (i.e., time, costs, and access to participants from other sites), we conducted an instrumental case study (Stake, 1995; Yin, 2017) at a comprehensive university located in central China. Multiple pre-service music teachers from the music school of the university were selected as the participants of the study. The study site is designated as one of the main music teacher training institutions in its province. On average, approximately 200 students graduate from the full-time 4 years education programme each year, and most then become primary- or secondary-level music teachers. As a well-established research method, case study offers researchers opportunities to obtain in-depth insights into the phenomenon of interest and to link prior theoretical propositions to the particular issue (Stake, 2005). In this study, we used an AR-assisted musical instrument learning app, *Simply Piano*, which, according to the historical ranking data documented by Qimai^1^, arose as a forerunner of its kind then in China. This app provides an AR-based way of learning the piano. Working with a piano or a keyboard, it utilises the acoustic sensor embedded in a mobile device to recognise the notes that a user plays and provides visualised real-time feedback on the screen. By augmenting real sound with AR technology, *Simply Piano* can provide spontaneous feedback such as helping users to know immediately if the notes and rhythm of their playing are correct. Further, this app provides piano courses from basic to advanced levels and practice pieces for various learning purposes.

### Participants

A convenience sampling strategy was employed to recruit participant for the study. All Years 3 and 4 students who were experienced piano players with Level 8 and above certificates accredited by China Musician Association and had some prior knowledge about AR were approached. We first contacted an associate professor from the school of music, who helped us to recruit participants. Invitations were sent through WeChat—a popular messaging and social media app in China (Montag et al., 2018). Finally, six female students agreed to participate in semi-structured, face-to-face interviews. Given the fact that more female than male students choose music education as their future career in China, this all-female sample was unsurprising. Their ages ranged from 22 to 24 years. With the growing demands for extra-curricular music lessons in China in recent years (Zhuang, 2015), all participants reported prior experiences (2–3 years) of delivering private piano lessons at the local city where the university is located.

### Procedure

Before data collection, the participants provided their written informed consent to participate in this study. All participants were informed of the study’s purpose and guaranteed that their participation in or withdrawal from the study would incur neither reward nor punishment.

The semi-structured interviews were conducted in a piano practicing room by an experienced qualitative researcher. Interview questions were derived from pertinent research (Waddell and Williamon, 2019). Each participant was interviewed twice. In the first interviews, participants were asked at what age they started learning piano and answered questions on their prior experiences/knowledge about AR and on their views about applying AR in music education. Next, each participant was allocated 2 h to get hands-on experience of *Simply Piano* installed on an iPad in a piano practice room. One researcher was present to provide guidance and to handle technical issues. In the second (follow-up) interviews, participants were asked about their perceptions of this AR-based piano learning app. Open-ended questions were employed to solicit participants’ experiences of interacting with *Simply Piano* and their views about employing this or similar apps in piano teaching and learning.

### Data Preparation and Analysis

When all interviews were completed, the second author transcribed all the interviews, and the first author checked the transcripts against the taped interviews to ensure the transcripts’ accuracy. During this step, participants’ personal information (such as name, year of study, and other personally identifiable information) was deleted, and each participant’s name replaced with a code (S1–S6). Next, drawing upon prior research on teachers’ perceptions of pedagogical use of technology (Liu et al., 2018; Yang et al., 2018), we performed a deductive thematic analysis. According to Braun and Clarke(2006, p. 84), this approach would allow researchers to retain focus on the pertinent aspects of interest. The step-by-step guide recommended by Braun and Clarke (2006) was followed. First, to get familiar with the data, each transcript was read through by both researchers. Then both researchers labelled the transcripts line-by-line with initial codes. These codes were further refined into subthemes to better capture the underlying ideas. The process was repeated for each transcript until no further themes could be interpreted from the data, and thematic saturation was reached. The disagreements that arose during the process of theme identification were resolved through discussion between the two researchers.

## Results

Taken together, the thematic analysis results provided a rich and deep understanding of the four pre-determined themes: (a) perceived benefits, (b) perceived disadvantages, (c) attitudes toward this app, and (d) intention to adopt AR-assisted music education. Information pertaining to each theme is presented below.

### Benefits

All interviewees opined that though they had limited knowledge of AR-assisted music education, they were impressed with the performance of *Simply Piano* and thought that linking acoustic instruments with mobile devices might benefit musical instrument instruction. Most pre-service teachers (except S3) indicated that the concept of AR technology could potentially positively influence beginner-level piano learners in three ways: (a) providing guidance and instant feedback, (b) keeping students engaged, and (c) affording more practice materials.

*I especially like the app’s capability to pick up the right note and show it on the screen immediately. It can definitely help students learn to read sheet music* (S1).

*The guide tutorials are easy to follow. Overall, I think this is a good piano learning app for beginners* (S6).

*The easy-to-difficult arrangement of lessons is well designed. It would keep students motivated to practice* (S4).

*Students would love this app. It is kind of like playing a game. They would find a lot of fun and a sense of achievement from this app* (S2).

*I used to spend a lot of time finding pieces that are popular. It is a pleasant surprise to find that the app provides a piano repertoire of various pieces that suit learners of different levels* (S5).

### Concerns

In contrast to these positive perceptions, some participants (*n* = 3) also expressed concerns regarding this approach. These concerns centred on the two subthemes of technical issues and content issues. First, two participants (S1 and S3) experienced technical malfunctions, as they reported:

*I noticed that if I started playing fast, the app may miss some notes. This is a bit annoying* (S1).

*The app is designed for beginners and cannot provide expert advice such as fingering and tempo. Those aspects are important for pianists. Using it will mislead students and likely be detrimental to their future development. You can only learn these from music teachers in person* (S3).

Further one participant (S5) stated, “*I am wondering whether there is a way for me to start from a higher level instead of the beginner level. It is a waste of time to start from the very basic stuff*”, indicating that the app could not provide the guidance she had expected.

### Attitudes Toward the App

When asked about their attitudes toward this approach, participants’ comments show varying degrees of approval, ranging from moderate approval (S5) to strong disapproval (S1 and S3).

*The experience of interacting with the app is enjoyable and I believe students can learn some basic stuff from it* (S5).

*This approach sounds great, but I think it is hard to apply in practice* (S1).

*I don’t think this app is good enough. I think it would be detrimental to students’ learning of advanced skills* (S3).

### Intention to Use the Approach

The overall responses from participants suggested they had either weak or no intention of employing the AR-based approach when they became teachers. As is pointed out by S4, this hesitancy may be first attributed to heavy influences from their personal learning paths, “*I can see its value but I would not [use it] because my own piano learning experience teaches me that teachers’ instruction is important.*” Next, expectations from parents may also be in the way, as one participant (S3) commented, “*It is unlikely that I will use this to teach piano. The parents would not like this approach. They hate digital devices and they would be afraid that I am being lazy and that their children would play instead of practicing*”. Lastly, the capacity of this approach at the current stage is also limited in their view, as reflected in the following comments.

*I may introduce this to my students so as to keep them engaged and find popular pieces to play. But the premise is that I will guide them* (S1).

*I think this way of teaching music is still at an early stage. It still has a long way to go* (S2).

*It is likely that I would use the app occasionally. Maybe I will use it to help me teach students read sheet music* (S5).

## Discussion

The purpose of this small-sample case study report was to provide a quick view of Chinese pre-service music teachers’ perceptions of AR-assisted musical instrument education. The findings indicate that despite some positive perceptions, participants doubted the efficacy of this instructional approach and exhibited weak intention to adopt it in their future classrooms.

In line with prior studies (e.g., Wise et al., 2011; Bauer and Dammers, 2016; Molloy et al., 2019), we found that these Chinese pre-service music teachers were generally aware of AR’s potential to help students learn to play musical instruments and keep them engaged. This awareness may be attributed to the large-scale promotion of ICT in the educational sector in China (China Internet Network Information Center, 2020) and the recent rise of ICT-based music learning tools, such as online music teaching/tutoring and smart musical instruments (Chen, 2019).

Meanwhile, echoing findings from similar research (e.g., Shoemaker and Stam, 2010), the study revealed that pre-service teachers have concerns regarding the approach. These concerns fall into two categories: (a) the inadequacy of the technology in handling certain situations, and (b) the lack of pedagogical design.

Contrary to prior research findings on music students’ willingness to use music technology, most participants in this study would be reluctant to adopt the approach in their teaching practices. Participants’ comments suggested that this reluctance was heavily influenced by external factors such as the traditional image of a good music teacher and parents’ expectations of music education in China. As discussed by Mu (1988) and Lin (2002), most of China’s music teacher training institutions adopt the traditional conservatory training model, focusing particularly on technical training (e.g., tone production, fingering, and weight transfer) rather than pedagogical skills. Thus, most pre-service music teachers tend to focus on technical proficiency.

Summing up, as technology increasingly reforms and reshapes the musical landscape, this study offers insights into the Chinese pre-service music teachers’ interaction with AR-assisted musical instrument learning from in-depth interviews with six pre-service teachers. Particularly, it draws our attention to their weak intention to use AR-assisted musical instrumental learning. One possible explanation for this phenomenon may be that though this approach did bring some promising benefits for beginning level musical instrument learners, given its performance in practice, these pre-service teachers mostly held it less favourable than in-person teaching mode and were thus reluctant to embrace it as a main teaching option. As is mentioned in Waddell and Williamon (2019), it seems that there is a still need to further align the development of software with specific learning outcomes so as to enhance the uptake of AR-assisted musical instrumental learning.

The limitations of the study need to be acknowledged. This report draws on only six female pre-service music teachers’ experiences of AR-assisted musical instrument learning with the intention of gaining a tentative, nuanced understanding rather than generalisation. Further quantitative research with a larger and more balanced sample is needed to reduce the sampling bias and further elucidate the pertinence of factors affecting music teachers’ acceptance of AR-assisted musical instrument learning. Also, it should be noted in this cross-sectional study, we only focused on one app. Future study can employ a wider range of technologies and adopt a longitudinal research design. Moreover, perspectives from music educators, in-service music teachers, and piano students about AR-assisted musical instrument learning are also important to help make sense of those findings in the future. Last but not the least, with this emerging trend in mind, more critical engagement with music education-related dimensions, such as the teacher-student relationship, may also be further explored.

## Data Availability Statement

The datasets presented in this article are not publicly available because the data contain the participants’ personal information. Requests to access the datasets should be directed to SY, hdysx@henu.edu.cn.

## Ethics Statement

Ethical review and approval was not required for the study on human participants in accordance with the local legislation and institutional requirements. The patients/participants provided their written informed consent to participate in this study.

## Author Contributions

BM: conceptualisation and methodology. SY: qualitative data analysis, resources, and funding acquisition. BM and SY: writing—original draft preparation, writing—review, and editing. Both authors contributed to the article and approved the submitted version.

## Conflict of Interest

The authors declare that the research was conducted in the absence of any commercial or financial relationships that could be construed as a potential conflict of interest.

## Footnotes

1 https://www.qimai.cn
